# A comparison of passive and active estimates of sleep in a cohort with schizophrenia

**DOI:** 10.1038/s41537-017-0038-0

**Published:** 2017-10-16

**Authors:** Patrick Staples, John Torous, Ian Barnett, Kenzie Carlson, Luis Sandoval, Matcheri Keshavan, Jukka-Pekka Onnela

**Affiliations:** 1 Department of Biostatistics, Harvard T. H. Chan School of Public Health, Boston, MA USA; 2 Department of Psychiatry, Beth Israel Deaconess Medical Center, Harvard Medical School, Boston, MA USA; 3 Division of Clinical Informatics, Beth Israel Deaconess Medical Center, Harvard Medical School, Boston, MA USA

## Abstract

Sleep abnormalities are considered an important feature of schizophrenia, yet convenient and reliable sleep monitoring remains a challenge. Smartphones offer a novel solution to capture both self-reported and objective measures of sleep in schizophrenia. In this three-month observational study, 17 subjects with a diagnosis of schizophrenia currently in treatment downloaded Beiwe, a platform for digital phenotyping, on their personal Apple or Android smartphones. Subjects were given tri-weekly ecological momentary assessments (EMAs) on their own smartphones, and passive data including accelerometer, GPS, screen use, and anonymized call and text message logs was continuously collected. We compare the in-clinic assessment of sleep quality, assessed with the Pittsburgh Sleep Questionnaire Inventory (PSQI), to EMAs, as well as sleep estimates based on passively collected accelerometer data. EMAs and passive data classified 85% (11/13) of subjects as exhibiting high or low sleep quality compared to the in-clinic assessments among subjects who completed at least one in-person PSQI. Phone-based accelerometer data used to infer sleep duration was moderately correlated with subject self-assessment of sleep duration (*r* = 0.69, 95% CI 0.23–0.90). Active and passive phone data predicts concurrent PSQI scores for all subjects with mean average error of 0.75 and future PSQI scores with a mean average error of 1.9, with scores ranging from 0–14. These results suggest sleep monitoring via personal smartphones is feasible for subjects with schizophrenia in a scalable and affordable manner.

## Patient monitoring: Smartphones can track schizophrenia-related sleep abnormalities

Smartphones may one-day offer accessible, clinically-useful insights into schizophrenia patients’ sleep quality. Despite the clinical relevance of sleep to disease severity, monitoring technologies still evade convenience and reliability. In search of a preferential method, a group of Harvard University researchers led by Patrick Staples investigated the validity of data collected via patients’ own mobile phones. The team, with a cohort of 17 schizophrenia patients, compared the quality of data produced by smartphone sensors and smartphone-delivered questionnaires to that of an in-clinic evaluation. The results significantly showed that smartphone monitoring could generate information that approached the accuracy of in-clinic assessments. The team noted some areas for improvement; however, this study provides convincing justifications for further research into this non-invasive, low-cost, scalable method to monitor the sleep quality of schizophrenic patients.

## Introduction

Sleep abnormalities are considered an important feature of schizophrenia^1^ and can be found in up to 80% of subjects.^2^ These sleep disturbances are of clinical importance given their relationship to symptom severity,^3^ psychotic relapse,^4^ premature mortality,^5^ and even suicide.^6^ Sleep disturbances may also represent an early marker of psychosis,^7^ and abnormal sleep architecture may serve as an intermediate phenotype of schizophrenia.^8^ Despite the clinical and research importance of sleep in schizophrenia, sleep monitoring remains a challenge. Self-report leads to overestimation of sleep duration,^9,10^ and while polysomnography remains the gold standard assessment tool, limited access^11^ and high costs restrict its clinical scalability and reach.

Actigraphy is the non-invasive monitoring of subjects, often via wearable sensors. Actigraphy has a rich history in psychiatric research,^12^ and it offers a partial solution to sleep monitoring. Even with modern sensors, actigraphy performs poorly compared to polysomnography in terms of sleep staging,^13,14^ but is nevertheless useful in collecting data on sleep timing and duration. Prior studies utilizing customized wearable sensors in schizophrenia have examined sleep duration, fragmentation, and efficacy.^15–18^ One study of 22 subjects wearing a customized actigraphy device suggested that reduced sleep efficiency positively correlated with next-day auditory hallucinations, and increased sleep fragmentation is positively correlated with next day paranoia and delusions.^16^ Other studies have also shown significant correlation between sleep disturbances detected by actigraphy and positive symptoms for schizophrenia^17^ and neuropsychological deficits.^19^

Despite these findings, actigraphy research suffers limitations in terms of reproducibility, scalability, and adherence. First, most actigraph research is done on customized devices, making it difficult to reproduce these studies and verify or build upon their findings.^16^ Second, most actigraphs remains expensive and deploying them in a large clinical setting is not a scalable approach, given their cost. Third, any wearable sensor suffers from adherence concerns, especially with longitudinal monitoring.^20^

There has been recent interest in the use of smartphones to monitor sleep across medical conditions including schizophrenia. Smartphones offer at least two approaches to monitoring sleep. First, it is feasible to infer objective measures of sleep through collection and analysis of passively gathered sensor data, such as accelerometry (similar to actigraphy), screen on/off logs, call logs, and ambient light/sound. Second, by offering ecological momentary assessments (EMAs) on the phone, it is also feasible to collect subjective measures of sleep in naturalistic settings, such as in the morning when recall of the previous night’s sleep is perhaps most accurate. As the prevalence of smartphone ownership continues to rise and as individuals with schizophrenia increasingly own and use these devices,^20,21^ smartphone-based sleep research offers a novel but readily available and easily scalable tool. In the U.S., smartphone ownership in the general population is now 77% (http://www.pewresearch.org/fact-tank/2017/01/12/evolution-of-technology/) and likely well above 50% for those with schizophrenia, although no official metrics exist.^22^ While some large-scale studies with over 8000 healthy subjects have already used smartphones to quantify global sleep patterns,^23^ their use in schizophrenia research is limited. Using the CrossCheck smartphone app, researchers derived sleep metrics from smartphone sensor data from 21 schizophrenia outpatients and found a positive correlation between sleep onset and hallucinations.^24^

In this paper, we further explore the potential of smartphones to monitor sleep in schizophrenia. A better understanding of this nascent monitoring tool would augment clinical knowledge about sleep, especially in long-term, large-scale monitoring settings, and as such it does not compete with but rather complements polysomnography, which remains the clinical gold standard. We compare smartphone-based monitoring to two commonly used clinical sleep monitoring methods, including self-reported EMAs done in real-time on the smartphone and gold standard clinical assessment with the Pittsburgh Sleep Questionnaire Inventory (PSQI).^25^

## Results

### Data quality

Among the 17 subjects enrolled, the mean age was 26.4, and 15/17 (88.2%) report their gender as male. Reported race includes six Caucasian, four Hispanic, three African American, two East African, one Caribbean, and one East Asian. At the time of analysis, over 90% of subjects used the Beiwe platform for over 6 weeks, and approximately half of all phone EMAs were co: see Supplementary Material Section 1 for more details. Additionally, passive data, or sensor and phone use data gathered without any interference with the user, was collected for these subjects throughout the study period. While several kinds of passive data are available for estimation of sleep duration, we focus our attention to the use of accelerometer data (see Methods for more details). Using accelerometer-based estimates and regular smartphone EMAs, we fit several statistical models to determine the relationship between PSQI, smartphone sleep EMAs, and accelerometer data. We also investigated the ability of smartphone data to predict PSQI scores.

**Fig. 1:**
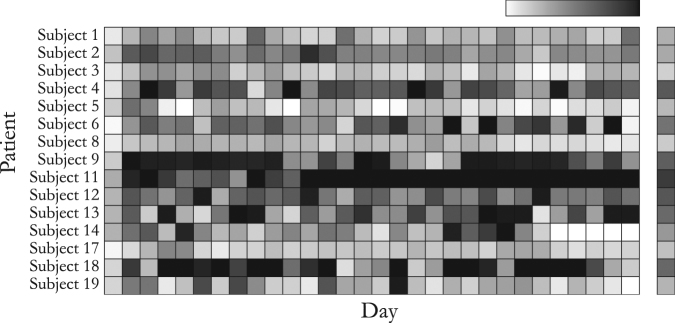
The amount of accelerometer data available for all subjects. High proportions of available data are shown in black, whereas low proportions of data are shown in white. The right-hand bar shows mean values within each subject. Across all subjects, mean proportion of accelerometer data is 46%, with a range of 2.1–89.1%

Figure 1 shows the amount of accelerometer data available for all subjects for 30 days. For each of our figures, all platform registrations, including test phones, were given anonymous Subject IDs ranging from 1–19. The wide variation in data available across subjects and days suggests that not all days will yield reasonable sleep estimates. For a brief investigation of data quality for specific subjects, see Supplementary Materials Section 2.

### PSQI and smartphone EMAs

**Fig. 2:**
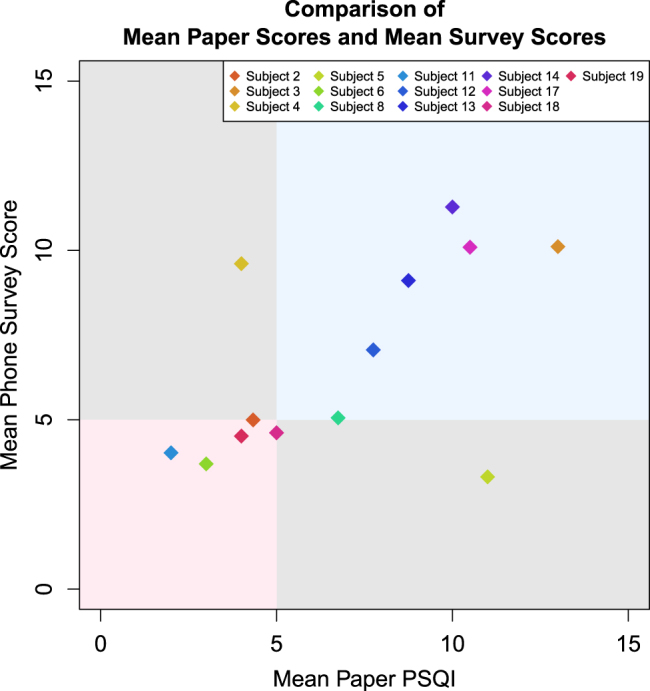
Comparison of mean paper scores and mean smartphone EMA scores scaled using cross-validated simple linear regression (*r* = 0.57, 95% CI 0.03–0.85). EMAs and passive data classified 85% (11/13) of subjects as exhibiting high or low sleep quality compared to the in-clinic assessments among subjects who completed at least one in-person PSQI

To investigate the relationship between the in-clinic PSQI scores and smartphone sleep EMA, we compare mean total PSQI scores to total mean phone sleep EMA scores for all subjects. A cross-validated simple linear regression model with mean phone-based EMA scores as the outcome and mean paper-based PSQI scores as the predictor, shown in Fig. 2, correctly classifies 85% (11/13) of subjects as exhibiting high or low sleep quality compared to the in-clinic assessments. One subject displays mean paper-based PSQI greater than the mean phone estimate, and one subject displays the opposite pattern.

### PSQI and smartphone accelerometer data

**Fig. 3:**
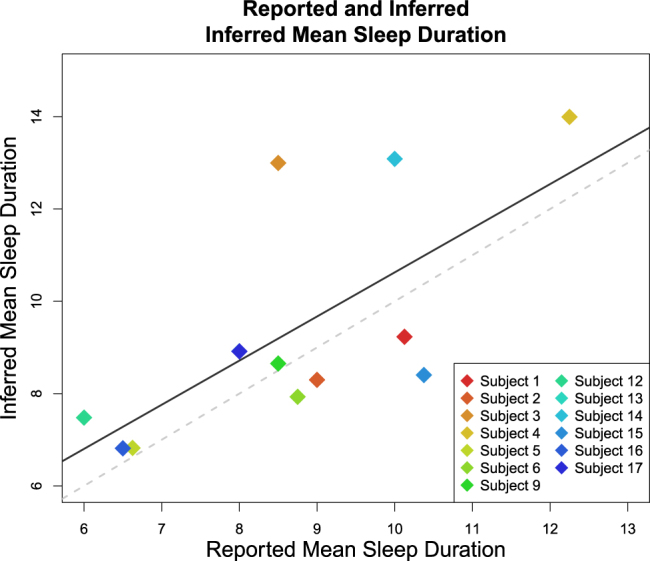
Comparison between mean reported sleep duration on the PSQI and inferred mean sleep duration from the smartphone accelerometer data, scaled using cross-validated simple linear regression, with inferred mean sleep duration as the outcome and the reported mean in-clinic sleep duration as the covariate. The solid line represents the regression line and the dashed line represents the unity line

We compared the accelerometer-based sleep estimates to the mean in-clinic subject self-reported hours of sleep, shown in Fig. 3. This figure suggests that our estimates of sleep based on passive smartphone data are correlated with smartphone EMA-based estimates of sleep, but the former tends to overestimate sleep duration. We find that the accuracy of accelerometer-based estimates as compared to phone EMA sleep durations are inversely related to accelerometer missingness (see Supplementary Materials Section 3). A direct assessment of the accuracy of our passive estimates of sleep duration and timing would require a comparison to contemporaneous polysomnography or actigraphy data, which was unavailable for this study.

### Correlates of sleep across entire study time for individual subjects

To find determinants of average PSQI scores across all subjects, we constructed a multiple linear regression model. Covariates included in the model, as well as their fit to the outcome of paper-based PSQI scores for each subject, are shown in Table 1. Although a linear regression shows a relationship between phone EMA responses and estimated duration of sleep (see Section 4 in Supplementary Materials), the regression relationship between mean or standard deviation in phone EMA-based sleep estimates compared to mean PSQI scores was not significant after accounting for other variables due to the high variability in the phone EMA-based sleep estimates for some subjects. This makes sense as the phone EMA questions are closely related to the PSQI while the passive data is used to estimate sleep duration, only one of many components comprising the PSQI score.

**Table 1:** Regression model fitting mean PSQI scores to available passive and EMA data for each patient

	Estimate	Standard error	*p*-value
Intercept	9.7865	2.0919	0.0095
Mean phone EMA answer	2.3479	0.4300	0.0055
Mean accelerometer-based estimated sleep duration	−0.1339	0.2019	0.5434
Mean accelerometer-based estimated Sleep variance	−0.0045	0.0089	0.642

Regression coefficients, standard errors, and *p*-values modeling the relationship between mean PSQI scores, mean estimated sleep duration (in hours), and estimated sleep variation. Only phone EMA scores significantly predict mean PSQI across subjects

Because mean smartphone EMA scores alone are significantly related to mean PSQI scores, we again fit mean EMA scores to mean PSQI scores using a simple linear regression model (*p*-value 0.0014). We used Leave-One-Out Cross-Validation (LOOCV), in which the model is fit on all subjects except one, and the omitted individual is used to test the prediction of the model trained on all other individuals. Fitting cross-validated sleep estimates from phone EMA scores to PSQI scores, we obtain a linear transformation relating daily phone EMA scores to estimated daily PSQI scores. In Section 5 of Supplementary Materials, we characterize these estimates and paper PSQI scores over time for each subject.

### Predictors of future PSQI scores for individual subjects

In-person and clinically assessed PSQI scores were ascertained for each subject ~1 month apart, which is the same timeframe for which a PSQI score is validated. In contrast, smartphone EMA questions are asked three times per week on the subjects’ smartphones and require no clinic visit or formal assessment. Passively collected accelerometer data was obtained continuously. This raises the possibility of using smartphone EMAs and passive data to predict PSQI scores. To estimate whether any of available phone sleep EMA or accelerometer data is predictive of PSQI scores while accounting for within-subject correlation, we constructed a linear mixed model. Predictors used include mean duration of sleep reported on the PSQI during the last clinical visit (if available), mean phone EMA scores since last visit, mean number of days for which sleep prediction data was available since the last visit, and passive data sleep estimates since the last visit. All data was weighted in this regression in proportion to the number of completed phone EMA responses since their last visit, taking into account the amount of data subjects provided via their phones. The results are shown in Table 2.

**Table 2:** Regression model fitting future PSQI scores to available previous data for each patient

	Estimate	Standard error	*p*-value
Intercept	1.1335	0.4366	0.0094
Mean passive estimate of sleep duration	0.0212	0.0430	0.6214
In-clinic reported mean sleep duration	−0.0642	0.0204	0.0017
Scaled mean phone EMA score	0.1218	0.0382	0.0014
Mean days of available passive data	0.3515	0.4728	0.4572

Estimated coefficients and their standard errors and *p*-values from a regression model fitting future PSQI scores to available previous data for each patient

**Fig. 4:**
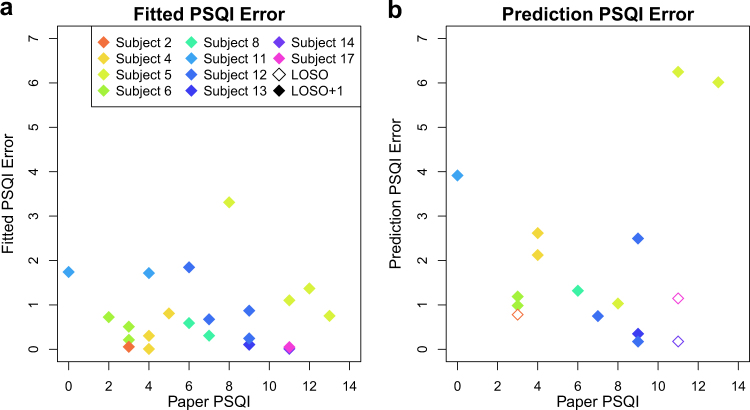
Mean average error in cross-validated prediction of future PSQI scores and model fit. Panel **a** shows the mean average error between predictors of PSQI using previous phone EMA responses and passive data, and the last measured PSQI score if available. Panel **b** shows the mean average error fitting PSQI score to previous data, with coefficients given in Table 2

Of the two significant predictors in this model, the mean phone EMA scores were the most predictive. This model was fit on all subjects, and the results are shown in the left panel of Fig. 4. The mean average error, a measure of how far off our predictions are from the actual PSQI, was 0.751, meaning that our prediction was within one point of the actual PSQI score.

To further assess the utility of our model, for each subject we created a new model only including data from other subjects (LOOCV). This corresponds to giving the Beiwe app to a new subject and using their phone EMAs and passive data to predict what their PSQI score will be based on patterns seen in other subjects. These results, along with the original model fit, are shown in the right panel of Fig. 4. Using cross validation, the mean average error (MAE), or mean absolute difference between actual PSQI scores and previous subject responses, gives a predictive mean average error of 1.925. This means that on average, phone EMA-based estimates of PSQI scores differ from proceeding paper-based PSQI scores by an average of 1.9, where scores range from 0–14.

## Discussion

In this study, we demonstrated the feasibility of using passively collected smartphone accelerometer data and smartphone-based EMA data from subjects with schizophrenia, related these measurements to in-clinic PSQI assessments, and demonstrated the potential of smartphone data to predict clinic-based sleep assessments. Unlike actigraphy or special sensors used to monitor sleep, there was nothing beyond the study app, Beiwe, that subjects had to install or use to record this data. While there are several limitations to our sensor-based results discussed below, the ability to collect this type of data with subjects’ smartphones at no direct cost in a simple and scalable manner is certainly one of the key advantages of our approach.

Comparing EMAs to in-clinic PSQI surveys across the entire study period for all subjects, we found that phone-based EMA scores alone were able to classify 85% (11/13) of subjects the same way as determined by an in-clinic PSQI assessment. We were also able to predict PSQI scores of subjects based only on EMAs and passive data across the evaluation period for all subjects with high accuracy (MAE 0.75). Combined, our results suggest that smartphone-based digital phenotyping is a feasible tool for studying and monitoring sleep for individuals with schizophrenia, and may be able to predict in-clinic sleep metrics.

The finding of strong correlation between PSQI scores and app-based ecological momentary assessments of sleep suggest a role for apps in monitoring sleep in schizophrenia. While prior pilot studies have explored the correlation of app-based ecological momentary assessments for positive, negative, and mood symptoms in schizophrenia,^22^ this paper focuses specifically on sleep. Our results suggest that the smartphone-based four-question sleep EMA that we utilized correlates strongly with the PSQI from which it was derived despite being administered in subjects’ daily lives on their personal smartphone. Offering subjects these brief smartphone EMAs may be a means to increase the availability and ease of sleep monitoring in schizophrenia.

The moderate correlation (*r*=0.69, 95% CI 0.23–0.90) between our passive estimate of daily sleep duration and PSQI reported sleep duration also suggests a role for passive data in characterizing sleep patterns in schizophrenia. This correlation may also be strengthened by averaging the measurements of these values over few months per subject, possibly leading to more stable mean estimates of both than those reported elsewhere.^10^ Smartphone passive data offers several advantages over ecological momentary assessment, especially for long-term adherence. While only approximately 50% of EMAs sent to the subjects were completed, the passive data estimates required no additional effort on the part of subjects. Prior studies in schizophrenia and bipolar disorder offer early investigations into use of smartphone-based accelerometer and suggest the feasibility in line with our results.^24,26^ However, these prior studies used passive data sleep results as one of many elements in larger multivariate models to predict symptom relapse, whereas our focus is on the nature of these sleep metrics themselves. This result is important as it suggests that correlation of passive data with gold standard clinical data alone does not necessarily imply clinical utility of that passive data, and that individual passive data streams alone may not be sufficient to characterize symptoms.

There are several possible reasons why the smartphone accelerometer data was statistically significantly correlated with both the PSQI and EMAs, yet the predictive power of passive data to estimate future PSQI scores was not statistically significant. Possible reasons include fundamental issues relating to actigraphy and phone sensors, variations in the subjects themselves, variation in subjects’ use of smartphones, and missingness in the passive data. First, even medical grade actigraphy device is not perfect, and while highly sensitive for detecting sleep (~90%), it suffers from low specificity (~50%).^27,28^ This means while 90% of actual sleep times are correctly estimated to be times of sleep, ~50% of estimated sleep times correspond to actual asleep. It is likely that smartphone-based actigraphy overestimates sleep, as accelerometer movement almost certainly subsides strictly before sleep and after waking. Adjusting for this bias in the absence of validation data is a difficult challenge, which we suggest for future work. Second, potential variation in sleep patterns of study subjects is influenced by differences in medication status, potential comorbid illnesses such as obstructive sleep apnea or restless leg syndrome, and many other factors that our study was not designed or powered to detect. Third, even if all subjects lacked variation in sleep patterns, it is likely that subjects vary in phone use. While smartphone-based estimates have face validity for detecting sleep in the use case where the phone is turned off right before sleep, put on a nightstand and not moved or accessed throughout the night, and accessed to turn off an alarm upon waking, there are many other use cases that may bias results. For example, if a subject has the phone in his bed, the accelerometer would detect vibrations from the bed and our algorithm may misinterpret such movement as reflecting an awake state. A fourth factor is that a significant amount of accelerometer data was missing. This may have been due to subjects turning off their phones, losing their phones, or other unexpected uses. This suggests that the advantage of unobtrusive sleep monitoring with smartphones may be more convenient than gold standard methods such as polysomnography, but may not be as accurate, limiting feasibility. As more data is accumulated, results may change. However, understanding these early results is necessary to ensure future modeling efforts are well informed.

In conclusion, our study suggests that smartphones are promising tools for assessing sleep in schizophrenia given their availability, facilitation of sleep-based ecological momentary assessment, and potential to gather passive data to estimate sleep duration. Further research with larger sample sizes will be necessary, but it is important that such research be done in a transparent and reproducible manner to allow future efforts to build off and learn from foundational efforts.

## Methods

The study was IRB approved by Beth Israel Deaconess Medical Center and followed the protocol outlined in an associated protocol paper.^29^ The goal of this study was to validate use of the Beiwe smartphone digital phenotyping platform for passive monitoring, ecological momentary assessments, and predicting sleep in schizophrenia. A limitation of earlier research conducted with smartphones is that many studies are carried out using proprietary sleep detection algorithms,^27^ which impede reproducible and transparent clinical investigation. At the time of analysis, we recruited 17 subjects with a clinical diagnosis of schizophrenia from a state outpatient mental health clinic. Subjects were eligible for the study if they owned a smartphone capable of running the Beiwe app (any Android or iOS phone running 5.0 or 9.0 or higher, respectively), their clinician at the clinic approved of their participation in the study, and they were able and willing to provide written informed consent. This three-month-long study was strictly observational, and the subjects downloaded and installed the Beiwe app onto their personal smartphone during the initial visit. It offered subjects smartphone EMAs of seven unique symptom questions three times a week. Subjects were asked to take a battery of psychiatry scales including the PSQI, MINI (psychosis section only), PHQ-9, GAD-7, and Warning Symptoms Scale at the initial visit, month one check in, month two check in, and month three (final) check in. Additionally, passive data (data collected without active subject participation such as accelerometer, GPS, screen use, and anonymized call and text message logs) was automatically collected for each subject, including measures of accelerometer activity, location determined from GPS sensors, social information from anonymized phone call and text message logs, and phone status of battery power. Subjects were compensated $25 for attending each study clinical visit, and they were paid $25 per month for use of their personal smartphone, although this compensation was not tied to app use. Subjects had the ability to call the study investigator for assistance if needed, although no additional coaching, therapy services, check in calls, or appointments were associated with this study. In keeping with the observational nature of the study, subjects did not get to view their data until after study completion. We captured three types of data: in-clinic, face-to-face gold standard questionnaires at monthly intervals, seven-question symptom EMAs administered on the phone three times a week, and constant passive collection of phone sensor and use data.

In addition to monthly in-clinic PSQI, each week subjects were asked to respond to the following four sleep questions as pop up EMAs on the smartphone according to the following prompt:

*In the last week, have you been bothered by the following problems?*

*Not at all* = *0, Sometimes* = *1, Often* = *2, Frequently* = *3*

- *Difficulty staying asleep*
- *Feeling tired*
- *Waking up too early*
- *Difficulty falling asleep*

We estimated the daily duration of sleep from each subjects' accelerometer data only; in Supplementary Materials, see Section 6 for statistical details on the estimation procedure and Section 7 for a discussion of how accelerometer data is used for estimation in this paper. In short, we expect that subjects would be expected to sleep with their phones resting in the vicinity of their bed. This lack of movement of the phone, detected by the accelerometer, has been used to estimate sleep duration in other studies^27^ and represents a simple variant of basic actigraphy.

Other potentially useful data streams for this purpose include GPS and power state. However, GPS might not be appropriate as some subjects may not leave the house with sufficient regularity to act as a signal of wakefulness. Additionally, power state is not gathered continuously, but rather only power state toggling between on and off are gathered. However, some values of this toggling are not recorded, and we are unaware at present time of reasonable means to impute values for these missing toggle points in the absence of validation data.

### Data availability statement

Data for this study will be kept on file per local IRB regulations. Although access to data in this study is restricted per study protocol^29^ due to subject identifiability, the Beiwe data collection and analysis platform are currently scheduled for public release late 2017, affording similar external validation by research teams. In addition, the configuration files specifying the data collection schedule for subjects in this study and the R code used for analysis are made available as supplementary files to this article.

## Electronic supplementary material

Supplementary Material
