# Supplementary material for: A comparison of passive and active estimates of sleep in a cohort with schizophrenia
Source: NPJ Schizophr. 2017 Oct 16;3:37. doi: 10.1038/s41537-017-0038-0 (PMC5643440; doi:10.1038/s41537-017-0038-0)
Supplement: Supplementary file 1 — Supplementary Material [file 41537_2017_38_MOESM1_ESM.pdf]

Supplementary Material

# 1 Survey Adherence

Figure 1 shows the proportion of completed smartphone surveys and the proportion of patients remaining in the study over time. The proportion of phone surveys completed appears reasonable, and increases slightly as the proportion of individuals remaining in the study decreases. This suggests that subjects who remain in the study for longer are also more adherent to completing phone surveys.

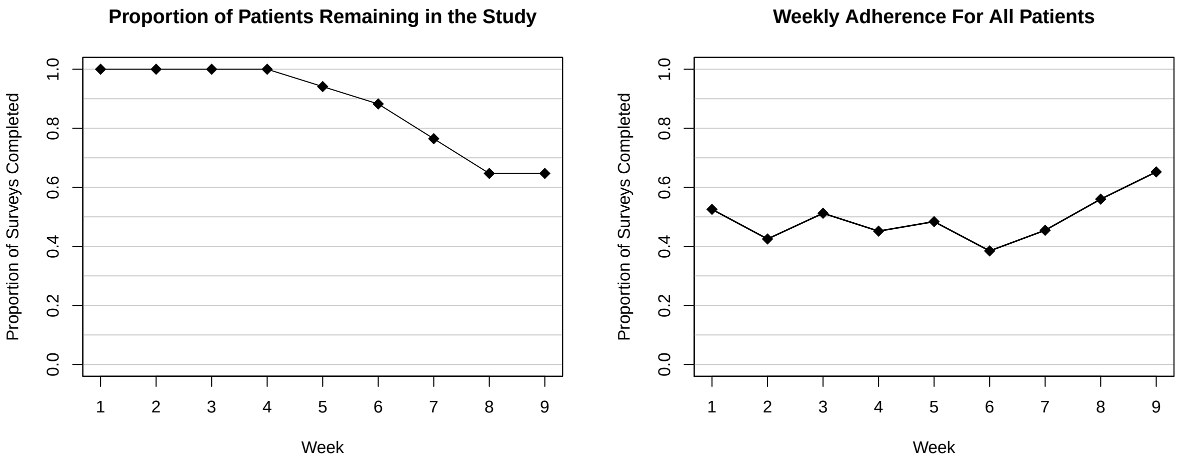


**Figure 1:** Adherence to smartphone surveys and to overall study. The left panel shows the proportion of patients that remain in the study. The right panel shows the average proportion of phone surveys completed among active subjects.

# 2 Accelerometer Data Quality

To show the variation in accelerometer data quality for specific individuals in the study, Figure 2 shows accelerometer activity for two sample subjects. White indicates no data, and colors from blue to red represent low activity to high activity, where activity is defined as the Euclidean norm of the standard deviation of force measured along the three accelerometer

axes. Subject 5 (left panel) shows little missingness, enabling sleep estimation, and tends to have low accelerometer activity near hours 10 and 18, indicating regular sleep. In contrast, Subject 6 (right panel) has more missingness, and does not show daily patterns in accelerometer activity.

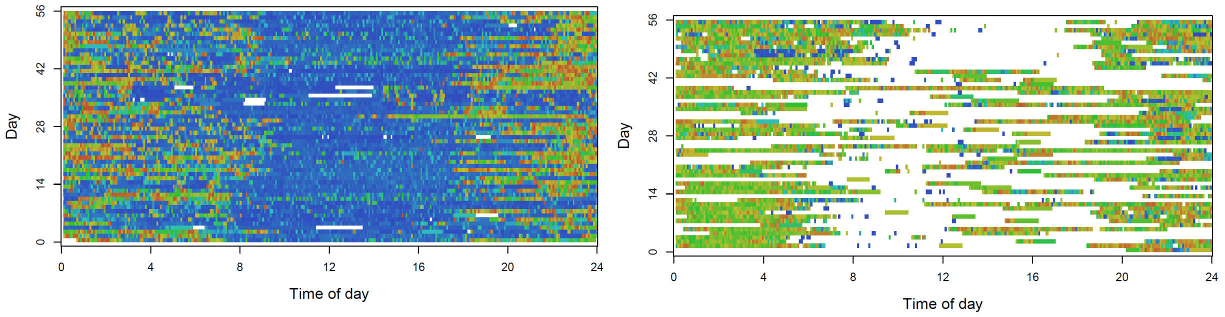


**Figure 2:** Accelerometer activity for Subjects 5 (left panel) and 6 (right panel). The x-axis shows the hour in the day, shifted ahead (forward) by 8 hours in order to center the period of low accelerometer activity in the plots. The y-axis shows accelerometer activity for each day where available. Low activity is shown in blue, whereas high activity is shown in red. Missing periods are shown in white.

# 3 Accelerometer-Based Sleep Duration Accuracy and Accelerometer Data Quality

To assess whether the accuracy of the accelerometer-based passive estimates of sleep duration depended on the amount of data available, we plotted the mean average error between these estimates and the subjects' EMA-reported sleep duration, with a single covariate of the amount of accelerometer data that was available for each subject as compared to the expected full amount of data (at least some data available for each 5-minute segment of each day). Figure 3 shows the relationship between these two quantities. While the number of subjects in this plot limit clear conclusions, it appears that data coverage below 30% is linked to much less accurate accelerometer-based sleep estimates. This suggests that investigators should attempt to ensure that data quality does not fall much below this threshold.

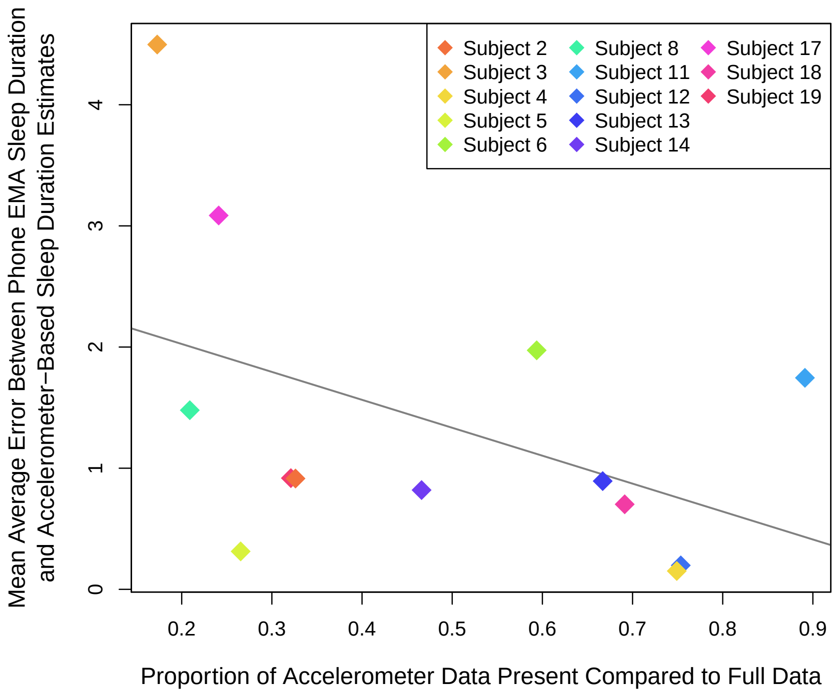


**Figure 3:** Comparison between inferred mean sleep duration accuracy and the amount of accelerometer data present for each subject for which data is available. The $x$ axis shows the proportion of accelerometer data present for each subject as compared to the expected full amount of accelerometer data, and the $y$ axis shows the mean average error between accelerometer-based sleep duration estimates and reported phone EMA sleep duration. The line shows a linear fit.

# 4 Smartphone Sleep Quality EMAs and Smartphone Accelerometer Data

To assess the relationship between mean accelerometer-based daily sleep estimates (in hours) and smartphone survey based sleep estimates (on a scale between 0-20), we created a linear model shown in Figure 4. The covariate for this model is a single phone survey response to one of the questions given in the Methods section, and the outcome is the estimated sleep duration on the day the survey response was recorded. While it is difficult to directly compare these two data sources, the negative correlation coefficient and the figure make intuitive sense as lower phone sleep scores, suggesting better quality sleep, are associated with longer sleep duration. While this simple regression is significant ($p$-value 4.9e-7), it does not account for within-subject correlation over time.

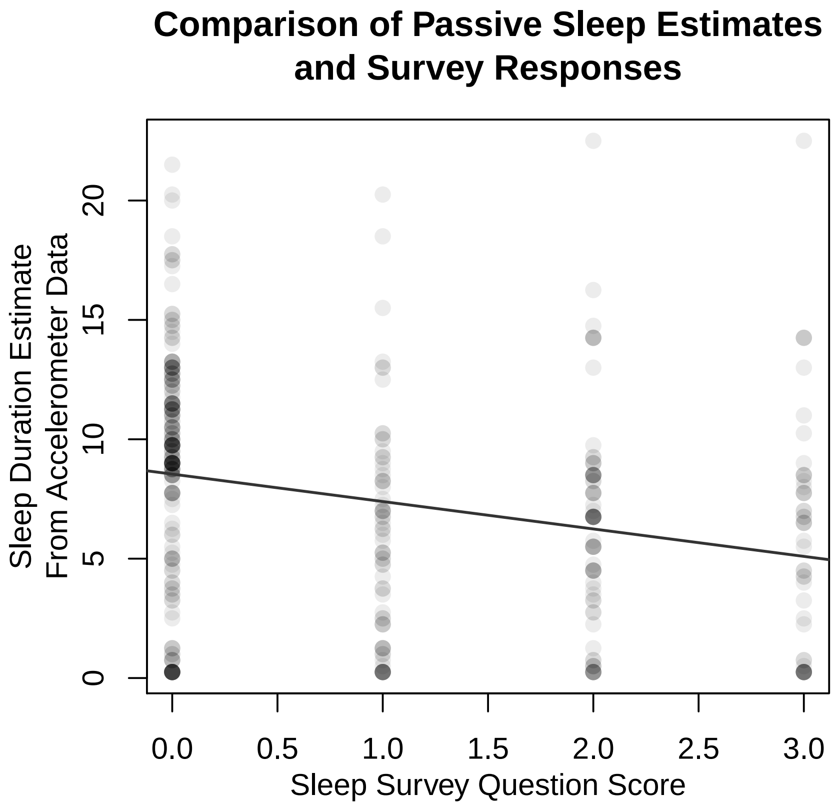


**Figure 4:** Comparison between inferred mean sleep duration from the smartphone accelerometer data and mean phone survey scores, scaled using cross-validated simple linear regression. Each dot represents a single survey response from a single patient. The *x*-axis shows a phone survey response to one of the questions listed in the Methods section. The *y*-axis shows the estimated sleep duration for the patient on the day when the survey question was answered.

## 5 Sleep Quality Over Time

Figure 5 shows estimated PSQI in all subjects for which longitudinal data is available over time, as well as paper-based PSQI scores and accelerometer-based sleep estimates. For several subjects, phone survey and paper estimates of sleep are reasonably correlated over time. Mean phone survey scores, and in-clinic PSQI scores are correlated over time, suggesting phone survey estimates of sleep are broadly indicative of sleep quality.

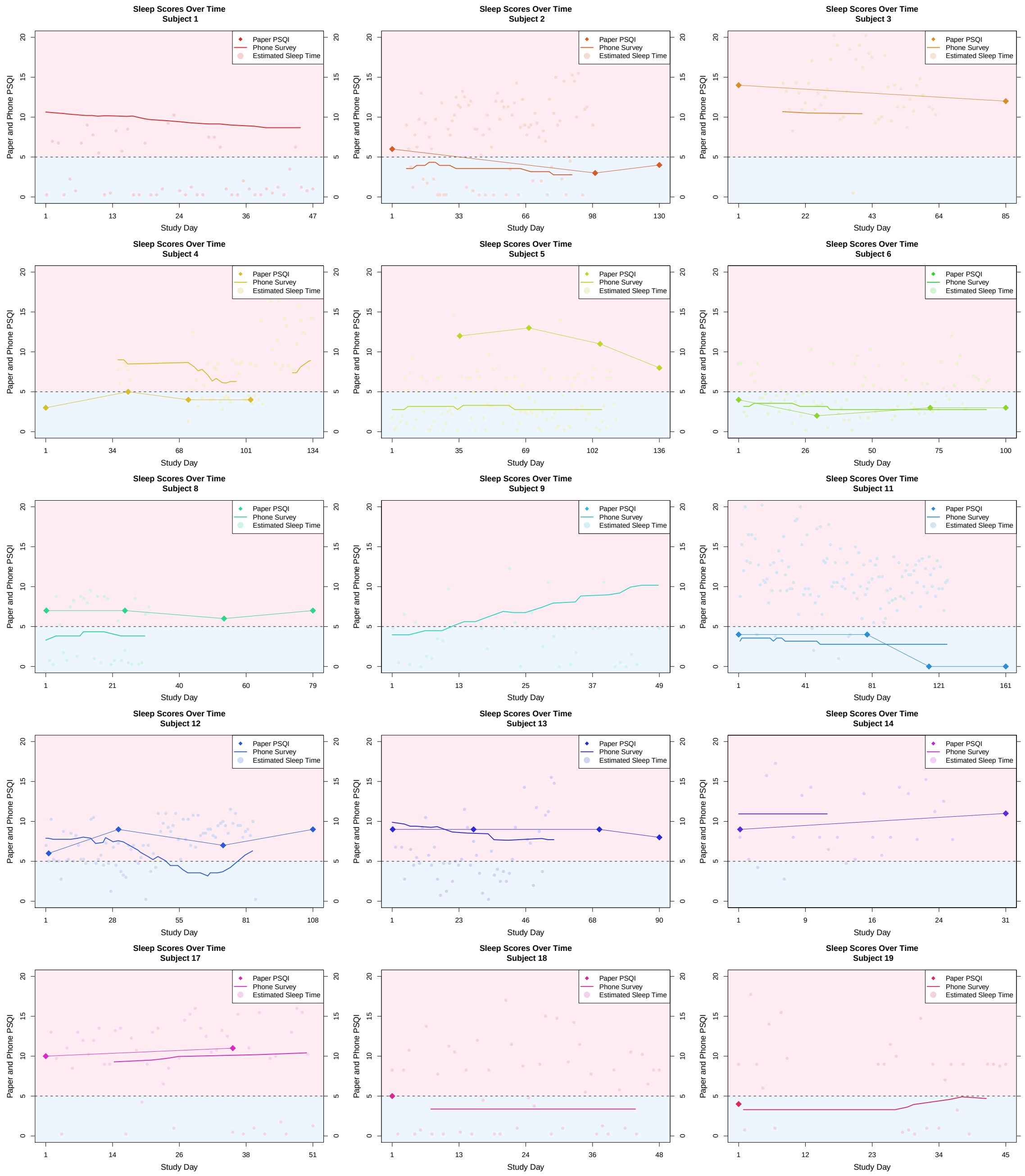


**Figure 5:** Estimates of adjusted mean phone survey sleep estimates, PSQI estimates within the clinic, and estimates of sleep for subjects across time.

# 6 Sleep Estimation Method

Notation. In this section, we outline the method we used to estimate sleep from accelerometer data. Let $i = 1, ..., D$ the days under study, $t$ be real daily time, and $\mathscr{T} = [0,\ T]$ be the daily interval, shifted so the sleep interval is approximately centered at $T/2$. We assume a single time $T^s$ indicates the onset of sleep, and $T^a$ indicated the onset of waking, which are both random variables, jointly written $\boldsymbol{T} = (T^s, T^a)$. These definitions exclude the possibility of naps and mid-sleep activity. We assume a univariate function of the data $Z_i(t)$ used to estimate the daily sleep interval is univariate and

indexed by day $i$ and daily time $t$. One candidate for this variable is accelerometer and actigraphy movement, screen events, or relevant bluetooth proximity. For a supervised approach, multivariate data may be fit to true sleep intervals, and the resulting model predictions may be used as the objective variable.

Identifiability Assumptions. We do not assume true outcome data is necessarily available, and make additional assumptions for identifiability. We assume *daily stationarity* (Assumption 1), or that the distribution of the objective variable is identical for each day. We also assume that the objective function is an interval function (Assumption 2), or that the only relationship between the distribution of the objective variable is a function of times to onset of sleep and waking.

$$Z_i(t) \underset{\text{iid}}{\sim} F_Z \qquad \forall i \tag{1}$$

$$Z_i(t) \mid \boldsymbol{T} \perp t \quad \forall t \tag{2}$$

Form of the Distribution. Let $F_T^s$ and $F_T^a$ be the distribution of the onset of sleep and wakefulness respectively. Similarly, let $F_Z^s$ and $F_Z^a$ be the distribution of $Z$ during periods of sleep and wakefulness respectively, and assume that corresponding probability density functions $f_Z^s$ and $f_Z^a$ exist. The probability of sleep can be shown to be $1 - F_T^s(t) + F_T^a(t))$, and the probability of wakefulness is $F_T^s(t) - F_T^a(t)$. Using the identifiability assumptions, the law of total probability, and Bayes's Theorem, it is straightforward to calculate the probability of the objective variable and the daily onset of sleep and waking:

$$\log \mathbb{P}(Z) = \sum_{i=1}^{D} \int_{\tau=0}^{T} \log \left\{ f_Z^a(Z_i(\tau)) - (f_Z^a(Z_i(\tau)) - f_Z^s(Z_i(\tau))) \cdot [F_T^s(\tau) - F_T^a(\tau)] \right\} d\tau \tag{3}$$

$$\begin{aligned} \log \mathbb{P}(\boldsymbol{T}_i|Z_i) = &\log f_T^s(\tau^s) + \log f_T^a(\tau^a) \\ &+ \int_{\tau=0}^{T} \log \left[ f_Z^s(Z_i(\tau))\mathbb{I}(\tau \in \mathscr{T}^s) + f_Z^a(Z_i(\tau))\mathbb{I}(\tau \in \mathscr{T}^a) \right] d\tau - \log \mathbb{P}(Z_i) \end{aligned} \tag{4}$$

Parametric Assumptions. Our current approach to estimate these distributions is to make parametric distributional assumptions, derive the maximum likelihood estimates for this parameter set, and calculate maximum likelihood sleep intervals conditional on these parameters. Specifically, we assume the objective function and onset to wake and sleep are normally distributed. The likelihood of the objective function and probability of the daily onset of sleep and waking is then defined as $\mathcal{L}(\boldsymbol{\Psi}|Z)$ and $\mathbb{P}(\boldsymbol{T}_i|Z_i, \boldsymbol{\Psi})$, respectively. We derived and solving the score equations for parameters and daily intervals as $\boldsymbol{\mathcal{U}}_j(\boldsymbol{\Psi}) = \frac{\partial \log \mathcal{L}(\boldsymbol{\Psi}|Z)}{\partial \Psi_j}$ and $\frac{\partial \log \mathbb{P}(\boldsymbol{T}_i|Z_i, \boldsymbol{\Psi})}{\partial T_i}$ respectively, as well as the Fisher information $\boldsymbol{\mathcal{I}}(\boldsymbol{\Psi})$ for confidence intervals and tests.

# 7 Data Used For Estimation

Although Beiwe gathers several types of data for potential usage in digital phenotyping, only accelerometer data is used to estimate sleep. For each day $i$ and time $t$ force is measured continuously in units of Earth's gravitational constant $g$

along three separate axes $x_i(t)$, $y_i(t)$, and $z_i(t)$. We combine each of these in blocks of 5 minutes, where for each block $j$ we define the observed objective function for this time $Z_i(t_j)$ these using the Euclidean norm of the variation along each axis:

$$\widehat{Z_i}(t_j) = \sqrt{\widehat{\text{Var}}(x_i(t_j)) + \widehat{\text{Var}}(y_i(t_j)) + \widehat{\text{Var}}(z_i(t_j))} \tag{5}$$
